# Age- and sex-specific differences in propofol consumption and extubation time: An analysis of a data set from an observational multicenter trial

**DOI:** 10.1097/MD.0000000000042176

**Published:** 2025-04-18

**Authors:** Cornelius Rahe, Barbara Schultz

**Affiliations:** a Department of Anesthesiology and Intensive Care Medicine, Hannover Medical School, Hannover, Germany.

**Keywords:** age-specific differences, anesthesia, EEG, emergence time, propofol dose, sex-specific differences

## Abstract

The objective of the present analysis was to investigate the effect of age and sex on propofol doses and emergence times in a dataset of propofol/remifentanil anesthetics. A total of 876 patients (339 men, 537 women; age: 18–87 years) with target electroencephalogram (EEG) stage D/E during maintenance of anesthesia and EEG stage D_2_/E_0_ at the end of propofol infusion were included (EEG monitor: Narcotrend; EEG in D/E range is characterized by delta activity [0.5–3.5 Hz]). Multiple linear regression analysis showed that total propofol dose (mean [standard deviation (SD)]: 6.81 (2.16) mg/kg/h) was significantly predicted by age (unstandardized regression coefficient *b* = −0.033, 95% confidence interval [CI]: −0.048 to −0.019, *P* < .001), sex (*b* = 1.542 [w (for women)], 95% CI: 0.573–2.512, *P* = .002), and interaction of age and sex (*b* = −0.022 [w], 95% CI: −0.041 to −0.004, *P* = .016). Propofol steady state dose (mean [SD]: 4.77 [1.74] mg/kg/h) was significantly predicted by age (*b* = −0.021, 95% CI: −0.036 to −0.006, *P* = .005), sex (*b* = 1.811 [w], 95% CI: 0.795–2.828, *P* < .001), and interaction of age and sex (*b* = −0.028 [w], 95% CI: −0.048 to −0.009, *P* = .004). For time to extubation (mean [SD]: 9.67 [4.51] mg/kg/h), age (*b* = 0.042, 95% CI: 0.010–0.073, *P* = .009) was a significant predictor, while sex (*b* = −0.655 [w], 95% CI: −2.785 to 1.475, *P* =.546) and interaction of age and sex (*b* = −0.011 [w], 95% CI: −0.051 to 0.029, *P* = .585) were not significant predictors. The administered remifentanil steady state dose (mean [SD]: 0.26 [0.12] µg/kg/min) did not differ significantly between men and women (*P* = .156) and decreased significantly with increasing age in men (*P* < .001) and women (*P* < .001). Age- and sex-associated differences in propofol requirements and a wide variation in propofol doses were observed. On average, women aged ≤40 years required comparatively high doses of propofol. The observations underline the importance of individually adapted anesthesia management, including monitoring of cerebral effects of propofol.

**Key Points:**

- Homogeneous data set with regard to exclusive use of propofol/ remifentanil for maintenance of anesthesia and with regard to EEG depth of anesthesia.
- Sex difference regarding propofol demand in patient groups ≤ 40 years.
- Propofol doses decrease with increasing age.
- High interindividual variability of propofol doses.

## 1. Introduction

Sex differences have been described for a variety of different groups of drugs used in anesthesia, including propofol, opioids, and muscle relaxants.^[1–5]^

Recently, Braithwaite et al (2023) published a systematic review and meta-analysis on the impact of female sex on anesthetic awareness, depth, and emergence.^[6]^ The authors concluded that available data on a relationship between sex and depth of anesthesia was heterogeneous and did not reveal clear sex-related differences. On the other hand, the authors found that women had a greater incidence of awareness under general anesthesia than men, and that emergence time, e.g., time to eye opening, was shorter in women than in men. Braithwaite et al did not describe age effects in their results.^[6]^

In another article published recently, Wasilczuk et al (2024) demonstrated that depth of anesthesia in adult mice on constant concentrations of inhaled anesthetics was dependent on the animals’ sex hormone status; age effects were not investigated.^[7]^ Correlations between sex hormone status and depth of anesthesia have also been demonstrated in female and male patients.^[8–11]^

Given the changes in sex hormones that occur in women and men throughout the course of life, we hypothesized that relationships between sex and depth of anesthesia may be age-associated. In a large data set of EEG-guided propofol/remifentanil anesthetics, we wanted to investigate propofol doses and emergence times in men and women, taking into account the patient’s age.

## 2. Materials and methods

### 2.1. Data set

The data set evaluated in this analysis comes from a multicenter observational study.^[12]^ The multicenter study was approved by the Federal Institute for Drugs and Medical Devices (BfArM) in accordance with the notification requirement.^[12]^ Results have already been published by Wilhelm et al, but these do not include any gender-related analyses.^[12]^ The analysis presented in this article focuses on comparing propofol doses and emergence times between men and women of different ages. This analysis was conducted after approval by the ethics committee of Hannover Medical School (No. 2910, March 22, 2002), and written informed consent was not required.

The multicenter study involved adult patients who underwent a surgical or diagnostic procedure in 1 of the 46 participating facilities (clinic or practice). The aim was to include anesthetics with a minimum duration of 1 hour. The decision to perform total intravenous anesthesia (TIVA) with EEG monitoring was at the sole discretion of the attending anesthesiologist. Apart from the restrictions as per the instructions for use for the drugs used, further selection criteria were deliberately not defined by the study protocol.^[12]^

**Figure 1.**
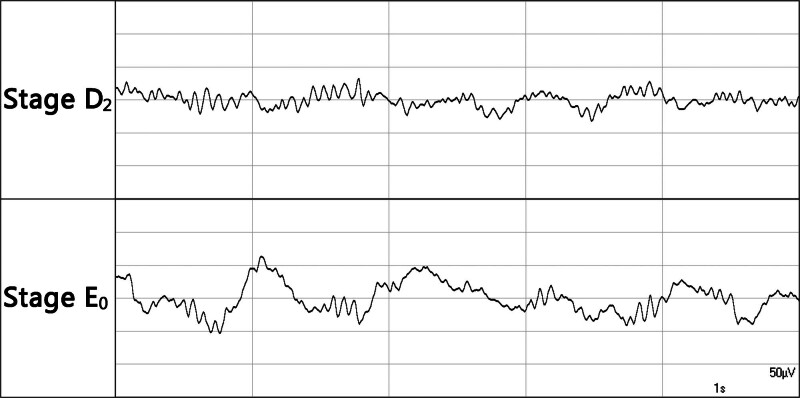
Examples of EEG stages D_2_ and E_0._ EEG = electroencephalogram.

The patients in the multicenter study received propofol for maintenance of anesthesia; the choice of analgesic administered and the dose were at the anesthesiologist’s discretion.^[12]^ Since the opioid administered during anesthesia could affect the emergence time at the end of anesthesia, all patients included in the retrospective analysis received remifentanil for maintenance of anesthesia. Remifentanil has a very short half-life.^[13]^ Propofol was given either as target-controlled infusion (TCI) or as manually-controlled infusion (MCI). During the steady state of anesthesia, EEG stage range D/E was targeted. All patients included in the retrospective analysis were in EEG stages D_2_ or E_0_ when the propofol infusion was stopped (EEG monitor: Narcotrend^®^, (MT MonitorTechnik, Bad Bramstedt, Germany)). EEG range D/E is characterized by delta waves (0.5–3.5 Hz). Two examples from sub-stages D_2_ and E_0_ are shown in Figure 1.

### 2.2. Endpoints and statistical analysis

The primary endpoint was the total dose of propofol (mg/kg/h), which included the amount of propofol given for induction and maintenance of anesthesia; additionally, for a subset of patients, i.e., patients with MCI, the maintenance dose of propofol (mg/kg/h) was available and was analyzed. The secondary endpoint was the emergence time, defined as time from stop of propofol infusion to extubation (min). In addition, the steady state dose of remifentanil (µg/kg/min) was analyzed in all patients.

Demographic data, propofol doses, extubation time, and remifentanil dose were compared between men and women by means of the Mann-Whitney test, with the rank-biserial correlation (*r*_B_) as effect size. Data was tested for normality with the Shapiro–Wilk test. For analyses relating to patient age, correlations and regressions were calculated separately for men and women. Multivariable linear regression served to identify predictors of the total dose of propofol, the steady state dose of propofol, and the extubation time; male sex was set as 0, female as 1, this is indicated as (w).

Cases where some data was missing were only excluded for analyses involving the missing data (missing values: height [men 5/women 5], body mass index [BMI] [men 5/women 5], time from stop of propofol to extubation [men 2/women 6], remifentanil [men 8/women 15]). The significance threshold was assumed to be *P* < .05. The data was analyzed using SAS (SAS Institute, Cary), version 9.3, 9.4 and JASP (JASP Team 2024), version 0.18.3.

## 3. Results

### 3.1. Demographics, drug doses, and time to extubation in men and women

The data set included 876 adult patients (339 men, 537 women). Table 1 compares demographic data for men and women. Men and women did not differ significantly with regard to age (*P* = .059) and BMI (*P* = .077). The men were significantly taller (*P* = <.001) and heavier (*P* = <.001) than the women. Table 1 also includes propofol and remifentanil doses and the time to extubation. The total propofol dose (including the induction bolus, all patients) was significantly greater in women than in men (*P* = .005), whereas the maintenance dose of propofol (without the induction bolus, patients with MCI, 186 men, 303 women) was not significantly different in women and men (*P* = .059). The median remifentanil dose administered during the steady state did not differ significantly between men and women (*P* = .156). The time from stop of propofol infusion to extubation was significantly longer in men than in women (*P* = <.001).

**Table 1:** Demographic data (age, weight, height, body mass index [BMI]), propofol doses, remifentanil dose, and time from stop of propofol infusion to extubation; median (25%-quantile, 75%-quantile).

	Men	Women	*r*_B_	*P*
n	339	537		
Age (yr)	52 (41, 63)	49 (39, 61)	0.076	.059
Weight (kg)	80 (74, 90)	69 (60, 80)	0.474	< .001
Height (cm)	177 (172, 181)	164 (160, 169)	0.786	< .001
BMI	25.88 (24.15, 28.37)	25.31 (22.57, 29.02)	0.071	.077
Propofol, total dose including induction dose, all patients (mg/kg/h)	6.41 (5.18, 7.63)	6.83 (5.37, 8.33)	−0.111	.005
Propofol, steady state dose, patients with MCI (mg/kg/h)	4.50 (3.50, 5.16)	4.76 (3.50, 6.00)	−0.102	.059
Remifentanil, steady state dose, all patients (µg/kg/min)	0.22 (0.20, 0.30)	0.25 (0.20, 0.30)	−0.057	.156
Time from stop of propofol to extubation (min), all patients	10 (7, 13)	8 (6, 12)	0.170	<.001

BMI = body mass index, MCI = manually-controlled anesthesia.

### 3.2. Propofol doses, extubation time, and patient age

Correlation analyses carried out separately for men and women showed that there was a negative correlation between total propofol dose and patient age in men (rho = −0.271, 95% confidence interval (CI): −0.367 to −0.169, *P* < .001) and women (rho = −0.404, 95% CI: −0.473 to −0.331, *P* < .001), that there was a negative correlation between propofol steady state dose and patient age in men (rho = −0.219, 95% CI: −0.352 to −0.077, *P* = .003) and women (rho = −0.371, 95% CI: −0.464 to −0.269, *P* < .001), and that there was a positive correlation between time from stop of propofol to extubation and patient age in men (rho = 0.176, 95% CI: 0.071–0.278, *P* = .001) and women (rho = 0.099, 95% CI: 0.014–0.182, *P* = .023).

**Figure 2.**
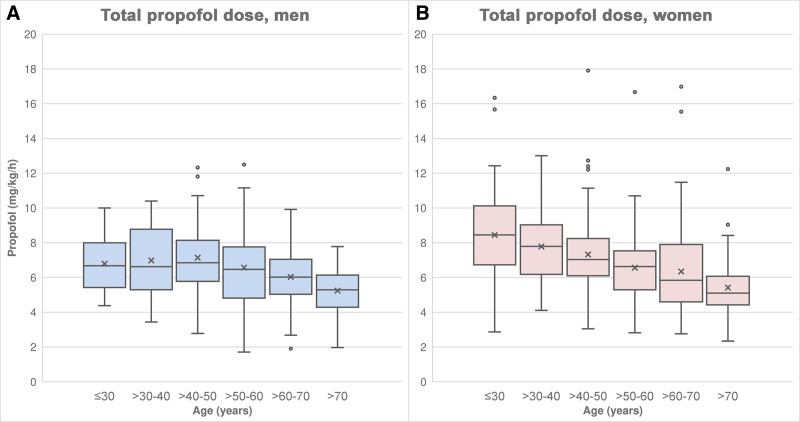
Total propofol dose (A) in men, (B) in women.

Figure 2A and B shows that the median total propofol dose in the age groups ≤ 30 years and >30 to 40 years was higher in women than in men. In the age groups ≤ 30 years (*r*_B_ = −0.445, *P* < .001) and >30 to 40 years (*r*_B_ = −0.209, *P* = .047), the difference between men and women was significant. In the other age groups, the difference was not significant (>40 to 50 years: *r*_B_ = −0.039, *P* = .641; >50 to 60 years: *r*_B_ = −0.006, *P* = .949; >60 to 70 years: *r*_B_ = −0.007, *P* = .942; >70 years: *r*_B_ = −0.003, *P* = .982).

Regression analyses between total propofol dose and patient age were performed separately for men and women. The regression coefficient *b* was −0.056 in women and −0.033 in men. According to the regression coefficients, the total propofol dose decreased per year by 0.033 mg/kg/h in men (*P* < .001) and by 0.056 mg/kg/h in women (*P* < .001).

**Figure 3.**
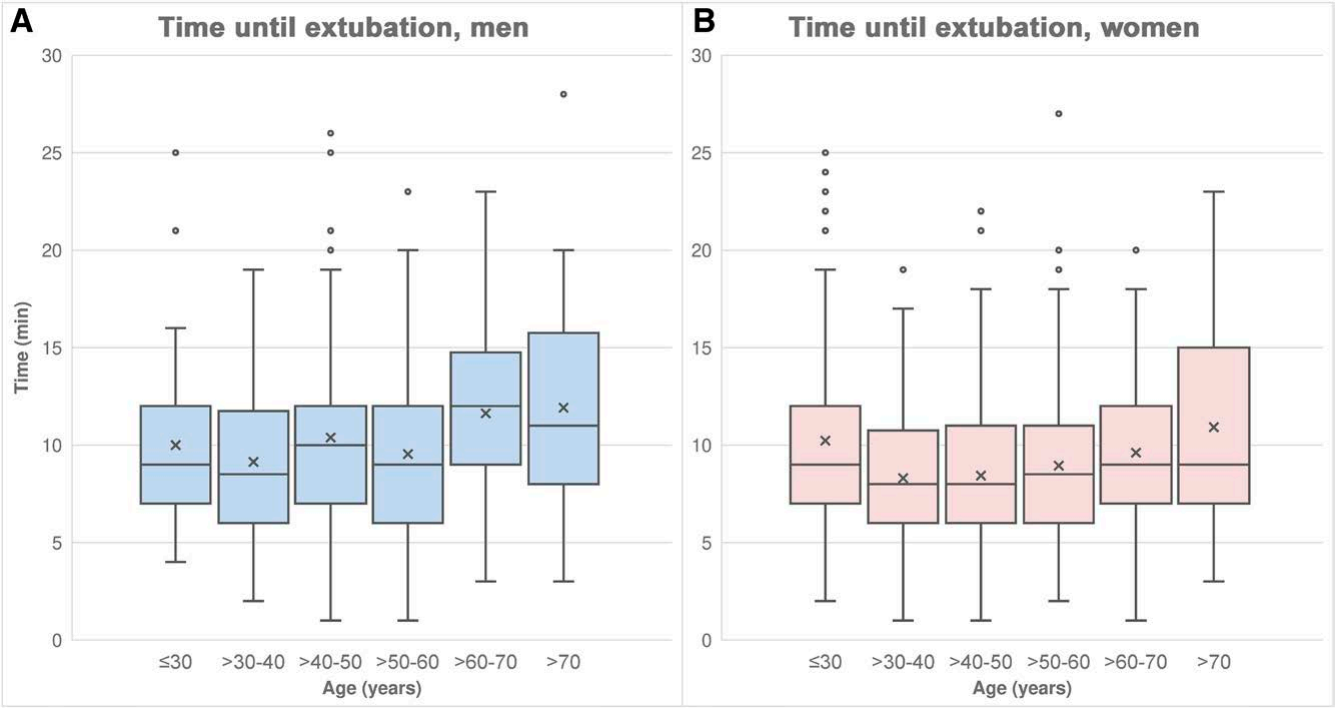
Time until extubation (A) in men, (B) in women.

From Figure 3 it is evident that, in men, the median time to extubation was longest in the age groups > 60 to 70 years and > 70 years. In the age group > 60 to 70 years, the time to extubation was significantly longer in men than in women (*r*_B_ = 0.305, *P* = .001). In all but 1 of the other age groups, the difference was not significant (≤30 years: *r*_B_ = −0.020, *P* = .874; >30 to 40 years: *r*_B_ = 0.100, *P* = .341; >40 to 50 years: *r*_B_ = 0.234, *P* = .005; >50 to 60 years: *r*_B_ = 0.084, *P* = .346; >70 years: *r*_B_ = 0.112, *P* = .359).

Regression analyses between extubation time and patient age were performed separately for men and women. The regression coefficient was greater in men (*b* = 0.042) than in women (*b* = 0.030). According to the regression coefficients, the increase in extubation time per year was 0.042 minutes in men (*P* = .013) and 0.030 minutes in women (*P* = .014).

### 3.3. Influence of age and sex on propofol doses and time to extubation

Multiple linear regression analyses were used to test whether age, sex, and the interaction of age and sex significantly predicted the dependent variables total propofol dose, propofol steady state dose, and time to extubation.

The total dose of propofol was found to be significantly predicted by age (*b* = −0.033, 95% CI: −0.048 to −0.019, *P* < .001), sex (*b* = 1.542 [w (for women)], 95% CI: 0.573–2.512, *P* = .002), and the interaction of age and sex (*b* = −0.022 (w), 95% CI: −0.041 to −0.004, *P* = .016). The adjusted R^2^ was 0.123.

The propofol steady state dose was significantly predicted by age (*b* = −0.021, 95% CI: −0.036 to −0.006, *P* = .005), sex (*b* = 1.811 (w), 95% CI: 0.795–2.828, *P* = .001), and the interaction of age and sex (*b* = −0.028 (w), 95% CI: −0.048 to −0.009, *P* = .004). The adjusted R^2^ was 0.129.

For the time to extubation, age (*b* = 0.042, 95% CI: 0.010–0.073, *P* = .009) was a significant predictor, while sex (*b* = −0.655 (w), 95% CI: −2.785–1.475, *P* = .546), and the interaction of age and sex (*b* = −0.011 (w), 95% CI: −0.051–0.029, *P* = .585) were not significant predictors. The adjusted *R*^2^ was 0.030.

### 3.4. Remifentanil steady state dose

Correlation analyses showed that the remifentanil steady state dose decreased significantly with increasing age in men (rho = −0.365, 95% CI: −0.455 to −0.268, *P* < .001) and women (rho = −0.290, 95% CI: −0.366 to −0.209, *P* < .001).

The influence of remifentanil dose, age, and sex on the propofol steady state dose was studied by means of multiple linear regression. In this data set, the remifentanil steady state dose (*b* = 1.157, 95% CI: −0.107–2.420, *P* = .073) was not a significant predictor of the propofol steady state dose, but age (*b* = −0.034, 95% CI: −0.044 to −0.023, *P* < .001) and sex (*b* = 0.341 (w), 95% CI: 0.036–0.646, *P* = .028) were significant predictors.

## 4. Discussion

In a large data set of EEG-guided anesthetics, which was homogeneous with respect to the opioid used, age and sex differences in propofol doses and emergence times were demonstrated. Women from the age groups ≤ 40 years, had the highest propofol consumption. Total propofol dose was significantly different in men and women aged ≤ 40 years. Propofol dose decreased with age in men and women. The emergence time until extubation increased significantly with age in men and women.

As early as 1993, Apfelbaum et al reported in a purely clinical study without EEG monitoring that age > 65 years and male sex were among the factors associated with delayed awakening after propofol anesthesia.^[14]^ In our own study, comparatively long emergence times were observed in men over 60 years of age.

Age-related changes in propofol requirements and emergence times can be explained by age-associated changes in the pharmacokinetics and pharmacodynamics of propofol. With increasing age, the body composition changes; with the same amount of propofol administered, higher propofol plasma levels are achieved in older patients than in younger patients.^[15]^ In addition, propofol clearance decreases with increasing age, which may be caused by a decrease in liver function or liver perfusion.^[16,17]^ An age-related change in pharmacodynamics in the sense of increased sensitivity to propofol in older people has been shown experimentally^[15]^ and could be caused by a loss of brain tissue (neuronal tissue), changes in receptor properties in the brain and possibly by changes in functional connectivity, i.e., communication between different brain regions.^[16]^

Several authors have observed sex-associated differences in propofol requirements and emergence times. Literature reports that women had shallower anesthetic stages than men at the same propofol dosage,^[18]^ and that women tended to require or required more propofol,^[19,20]^ or required a propofol TCI concentration that was significantly higher than in men to maintain a defined anesthetic EEG stage.^[21]^ However, there are also studies in which no difference was found between the sexes in terms of propofol requirements in patients with EEG-guided anesthesia.^[17,22]^ Several authors reported that emergence times after propofol anesthesia were shorter in women than in men. This applied to pure propofol sedation,^[19]^ combinations of propofol administration and spinal anesthesia,^[23]^ as well as propofol anesthesia with different opioids, such as alfentanil,^[24]^ fentanyl,^[25]^ and remifentanil.^[20,26,27]^ Büttner et al (2010) found no difference in emergence times.^[21]^

Men and women differ in their body composition. The fat content is higher in women and the water content is lower. This means that the volume of distribution (defined as the ratio between plasma concentration and the amount of drug in the body) of lipophilic drugs such as propofol is greater in women than in men and therefore the plasma concentration is primarily lower.^[1,3]^ However, in the steady state of anesthesia, elimination is more significant than the volume of distribution.^[3,22]^ Propofol is mainly metabolized in the liver.^[28]^ In a study by Loryan (2012), the concentration of CYP2B6, a degradation enzyme of propofol, was 1.9-fold higher in the liver of women than in men (*P* = .035).^[29]^ Loryan (2012) and Choong (2013) found significantly higher concentrations of glucuronidated propofol in women than in men.^[29,30]^ According to Choong (2013), this suggests that, compared to men, more rapid propofol metabolism may occur in women—a factor that may contribute to differences in propofol anesthesia efficacy between male and female patients.^[30]^ Apart from studies that describe pharmacokinetic differences, there are studies that suggest that the sensitivity of the female and male brain to propofol differs, but the results are not consistent.^[23,24]^

In our study, a significant difference in propofol dose was only found between women and men of the age groups ≤ 40 years. There was no significant difference in the other age groups of older patients. This suggests that effects of sex hormones may have contributed to the difference in dose. There are reports in literature on correlations between sex hormone levels and depth of anesthesia.^[7–11]^ In the course of life, the concentrations of sex hormones change in both sexes. In women, the menopause, which occurs between the ages of 45 and 60,^[31]^ is caused by a reduction in the secretion of estrogen and progesterone.^[32]^ Progesterone and its metabolites in particular have hypnotic effects, while estrogen has the opposite effect.^[8,9]^ Women’s testosterone levels decrease with age, but as women’s testosterone levels are about ten times lower than men’s, the effects of lower testosterone levels during aging may be more pronounced in men.^[33]^ In men, a gradual decline in testosterone can be observed, which begins at around 20 to 30 years of age and continues until death.^[33]^ Obesity and comorbidities can contribute to the decline in testosterone in men.^[34]^ Experiments in adult mice have shown that the administration of testosterone increased sensitivity to inhalation anesthetics, but the authors did not investigate age effects.^[7]^

In the introduction to this article, we made reference to Braithwaite et al (2024), who conducted a meta-analysis on the impact of female sex on anesthetic awareness, depth, and emergence.^[6]^ Without describing age effects in their results, the authors concluded that the data on the association between sex and depth of anesthesia is heterogeneous and does not show clear sex differences.^[6]^ Our analysis suggests that it is important to consider patient age when addressing this question.

A considerable variation in propofol doses was observed in the analyzed data set. In addition to the investigated factors age and sex, there are numerous other factors that can influence propofol requirements during anesthesia. These include, for example, alcohol consumption,^[35,36]^ drug abuse,^[37]^ cigarette smoking,^[38]^ anxiety,^[39]^ and concomitant diseases.^[40,41]^ In the analyzed data set, all patients received remifentanil as short-acting analgesic; the doses per kg of body weight per minute for men and women were not significantly different.

One aim of general anesthesia is to put patients into a state of unconsciousness for the duration of the surgical procedure. Unintentional awareness during anesthesia can have a traumatizing effect on patients and can have psychological consequences in the form of post-traumatic stress disorder.^[42]^ In a retrospective analysis of adverse anesthetic events, 77 % of patients affected by intraoperative awareness with recall were female. Of these, 89 % were younger than 60 years.^[42]^ Intraoperative connected consciousness without recall is characterized by patients’ responding to prompts intraoperatively without remembering this postoperatively. It is reported that in studies on connected consciousness after intubation, the incidence was higher in younger adults than in older adults.^[43,44]^ Among younger adults, women were more than twice as likely as men to experience connected consciousness, although the amount of propofol (mg/kg) given to women and men before intubation was not significantly different.^[43]^ These results are consistent with the observation from our own study that younger women required particularly high doses of propofol compared to men of the same age and compared to older patients of both sexes.

## 5. Strengths and limitations

A strength of the present analysis is that the analyzed data set is large and covers a wide age range, comprising almost 900 patients aged between 18 and 87 years. The dataset is homogeneous in terms of the opioid used, i.e., remifentanil, and the targeted EEG range during maintenance, i.e., stages D and E, which are characterized by delta activity (0.5–3.5 Hz). All patients were in EEG stage D_2_ or E_0_ when the propofol infusion was stopped, i.e., the starting point for emergence was similar in all patients.

The data is mainly from adult German Caucasian patients. With regard to data homogeneity, this is a strength, but with regard to generalizability of the results, it may be a limitation. As this is a reanalysis of data from a completed study, parameters that might have been of interest, such as sex hormone concentrations, were not available.

## 6. Conclusions

The observation of age- and sex-associated differences in propofol requirements as well as the observation of the wide variation in propofol requirements underline the importance of individually adapted anesthesia management, including monitoring of cerebral effects of propofol during anesthesia.

## Acknowledgments

We thank PD Dr. Ulrich Grouven for statistical advice.

## Author contributions

**Conceptualization:** Barbara Schultz.

**Data curation:** Cornelius Rahe, Barbara Schultz.

**Formal analysis:** Cornelius Rahe, Barbara Schultz.

**Methodology:** Cornelius Rahe, Barbara Schultz.

**Visualization:** Barbara Schultz.

**Writing – original draft:** Cornelius Rahe, Barbara Schultz.

**Writing – review & editing:** Cornelius Rahe, Barbara Schultz.
